# Symptom profiles and illness course among Anabaptist and Non-Anabaptist adults with major mood disorders

**DOI:** 10.1186/s40345-016-0062-4

**Published:** 2016-10-12

**Authors:** Kelly E. Gill, Stephanie A. Cardenas, Layla Kassem, Thomas G. Schulze, Francis J. McMahon

**Affiliations:** 1 Human Genetics Branch, Section on Genetic Basis of Mood and Anxiety Disorders, National Institute of Mental Health Intramural Research Program, National Institutes of Health, New York City, USA; 2 Department of Psychology, The Catholic University of America, 620 Michigan Ave NE, Washington, D.C., 20064 USA; 3 Institute of Psychiatric Phenomics and Genomics (IPPG), Medical Center of the University of Munich, Munich, Germany

**Keywords:** Depression, Mania, Bipolar disorder, Amish, Mennonite, Alcohol, Head injury, Concussion

## Abstract

**Background:**

Anabaptists comprise large and growing Amish and Mennonite populations with a unique genetic heritage and cultural background. Little is known about the symptoms and course of major mood disorders in Anabaptists. Even less is known about the impact of potential moderators on symptom severity and course.

**Methods:**

A sample of Amish and Mennonite participants with bipolar, recurrent unipolar, or schizoaffective bipolar disorder (*n* = 155) were systematically evaluated with a well-validated instrument. Cases were compared with non-Anabaptist participants (*n* = 155) matched for age, sex, and psychiatric diagnosis and evaluated by the same methods.

**Results:**

Despite substantial cultural differences, the profile of manic and depressive symptoms during illness episodes did not significantly differ between the two groups. Alcohol use disorder (AUD) was significantly less frequent among Anabaptists, and was associated with more major depressive episodes and more hospitalizations for major depression in Anabaptist, but not non-Anabaptist participants. Lifetime history of head injury showed a trend toward association with more episodes of major depression in both Anabaptist and non-Anabaptist groups that did not withstand multiple test correction.

**Conclusions:**

The presentation of a highly heritable psychiatric illness such as bipolar disorder does not differ in cases drawn from genetically unique Anabaptist populations. However, alcohol comorbidity, head injury, and their effects on illness course suggest some differences that deserve further investigation.

## Background

As psychiatry enters the age of genomic medicine, interest in genetically isolated populations is growing once again. Each genetically isolated population represents an unique “experiment of nature,” characterized by unusual gene frequencies, increased rates of homozygosity, and a greater prevalence of some otherwise rare inherited diseases (McKusick et al. 1964). A recent simulation study of genetic isolates (Zuk et al. 2014) illustrated how otherwise rare alleles that influence disease risk may rise to frequencies large enough to be detectable by genome-wide association studies. Here we present the first clinical results from an ongoing study of bipolar disorder among genetically isolated Anabaptist populations, known as the Amish Mennonite Bipolar Genetics study, or AMBiGen (Hou et al. 2013). The ultimate aim of AMBiGen is to identify alleles conferring substantial risk for bipolar disorder and related conditions.

Anabaptists include such well-known groups as the Amish and Mennonites, who originated in Western Europe, as well as several less well-known groups of varied ancestries (e.g. Hutterites, Schwarzenau Brethren). Some Anabaptists who migrated to North America in the early 1700s have opted to remain separate from the general population, marrying within their traditional communities and raising large families. They suffer from high rates of some otherwise rare diseases (McKusick 1980) and may also have an increased risk of mood disorders (Egeland and Hostetter 1983).

Tightly knit communities, a traditional agrarian lifestyle, and distinct religious beliefs distinguish many Anabaptists from the surrounding population (Brewer and Bonalumi 1995). Among the Amish and other traditional (“plain”) Anabaptists, for example, men commonly work as farmers, carpenters, or other craftsmen, while women take care of the household and the children (Kreps et al. 2004). Children typically begin working for the family business at age 14, and their formal education ends after the eighth grade (Kreps et al. 2004). Most do not use electricity, communicate by telephone, or drive motor vehicles at home (Weyer et al. 2003). They follow plain dress codes and grooming habits, which they believe reflect a rejection of vanity and materialism (Brewer and Bonalumi 1995). Daily life is centered on the home. Low rates of divorce and contraceptive use leads to large families (8–10 offspring), and multiple generations may share the same household or live as neighbors.

Some of the distinctive aspects of Anabaptist lifestyles may decrease vulnerability to develop certain mental illnesses. Potential protective factors include increased religiosity (Smith et al. 2003), increased social support from family, friends, and community members (Johnson et al. 1999), and lower rates of alcohol and substance use than in surrounding communities (Trimble 1994). However, it remains unclear to what extent lifestyle differences characteristic of Anabaptists affect the risk of mood disorders, illness course, or symptom severity.

A series of studies have already provided a detailed description of bipolar disorder and related conditions in the Old Order Amish of eastern Pennsylvania (Egeland and Hostetter 1983; Egeland et al. 1983; Hostetter et al. 1983). The Amish Study of Major Affective Disorder began in 1976 and examined Amish individuals with mood disorders, along with family members (Egeland et al. 1983). Overall, 71 % of the individuals studied had a major mood disorder: 34 % were diagnosed with bipolar I or bipolar II disorder, and 37 % were diagnosed with unipolar depression (Egeland et al. 1983). Diagnoses in these individuals were found to be valid and reliable, although there were expected cultural factors that influenced assessment of some symptoms (Egeland et al. 1983; Hostetter et al. 1983). Further research on this group included the search for genetic markers (Egeland et al. 1987; Ginns et al. 1998; Kelsoe et al. 1989), and studies of prodromal symptoms of mood disorders (Shaw et al. 2005). While the Amish Study of Major Affective Disorders provided the first and most detailed account of mood disorders in this population, the absence of a non-Amish comparison group precluded any direct comparisons of symptoms or course of illness.

The present study examined the symptoms and course of major mood disorders in Anabaptists ascertained from several communities in the United States. Comparisons were performed with a set of age and sex-matched cases assessed with the same instrument, but ascertained from the general (non-Anabaptist) population (Potash et al. 2007). We also examined potential moderating variables affecting the course and severity of mood disorders in both the Anabaptist and non-Anabaptist groups we studied.

## Methods

### Sample and procedures

Data pertaining to the Anabaptist group (*n* = 155) were collected over the course of 6 years (2009–2015). Participants were mainly recruited through clinical settings in Pennsylvania, Indiana, and Ohio. Some participants responded to advertisements placed in publications targeting Anabaptist households or were referred from others in the community who had knowledge of the study. The comparison group (here called “non-Anabaptists”) was drawn from 2897 individuals with a major mood disorder previously ascertained from 1991 to 2002 by the National Institute of Mental Health (NIMH) Genetics Initiative and collated in the Bipolar Disorder Phenome Database (Potash et al. 2007).

Clinical symptoms in both groups were assessed by direct interview with the Diagnostic Interview for Genetic Studies (DIGS), a widely used instrument with established reliability (Nurnberger et al. 1994). The DIGS is a semi-structured assessment of major depression, mania, psychosis, alcohol/drug abuse and dependence, suicidal behaviors, and anxiety disorders. The DIGS assesses symptoms over the lifetime, as well as for the most severe episodes of major depression and mania. Final diagnoses were assigned in a best estimate procedure by at least two clinicians who reviewed the DIGS along with information from family informants and available medical records.

Each case in the Anabaptist sample was matched on age (±3 years), gender, and DSM-IV diagnosis with a case drawn randomly from the Phenome Database using SPSS (v. 22). In both the Anabaptist and Non-Anabaptist groups, 69 % (*n* = 107) were diagnosed with bipolar I, 11 % (*n* = 17) with bipolar II, 8 % (*n* = 12) with major depression (recurrent), 9 % (*n* = 14) with schizoaffective bipolar, and 3 % (*n* = 5) with single episode major depression. This matching procedure was designed to minimize differences in clinical course and symptoms that could be attributable to the matched variables.

### Statistical analyses

All statistical analyses were performed using SPSS (v. 22). Selected demographic and lifetime psychiatric history variables were assessed using *χ* ^2^ or *t* tests, as appropriate, with Bonferroni correction for 21 tests (*α* = 0.002 was declared significant). Individual symptoms reported during the most severe episodes of mania and major depression were compared using *χ* ^2^ tests, with Bonferroni correction for the 29 variables tested (*α* = 0.001 was declared significant). Dependent variables chosen to give a picture of the lifetime severity and course of the mood disorder included: age at first major depression, number of symptoms during the most severe episode of major depression, number of hospitalizations for major depression, number of major depressive episodes, and frequency of major mood episodes (calculated as the total number of manic or major depressive episodes per year of illness) (Fisfalen et al. 2005). The dependent variables were converted into standardized *Z*-scores for all analyses.

Predictor (independent) variables included: group (Anabaptist vs. Non-Anabaptist), reported head injury, and presence of an alcohol use disorder (DSM-IV alcohol abuse or dependence). There were too few Anabaptist cases with other comorbidities to support any powerful comparisons. A history of admission to a psychiatric hospital was included as a covariate, since the majority of the Anabaptist sample was recruited from hospital settings, but many of the non-Anabaptists were not. Categorical predictor variables were analyzed using two-way analysis of covariance (ANCOVA) with Bonferroni correction for 3 × 5 = 15 tests (*α* = 0.003).

## Results

### Demographics and comorbidity

As expected, there were demographic differences between the Anabaptist and Non-Anabaptist cases (Table 1). Anabaptists had more children and fewer years of education. Anabaptists reported a significantly higher rate of major role impairment due to their illness and had higher rates of psychiatric hospitalization and psychotropic medication use.

**Table 1:** Demographic and clinical characteristics of Anabaptists and Non-Anabaptists

	Anabaptist	Non-Anabaptist	Analysis
	*n* (%)	*n* (%)	*χ* ^2^
Married	96 (61.9)	72 (47.7)	6.28
Medical illness*	42 (27.5)	75 (48.7)	14.69
Ever treated	150 (98.0)	144 (93.5)	3.89
Unable to function*	132 (90.4)	118 (77.1)	9.63
Ever Hospitalized*	118 (82.5)	79 (52.7)	29.61
Medication	138 (96.5)	134 (87.6)	7.90
Polarity*			15.54
1st mania before 1st depression	59 (38.1)	35 (22.6)	–
1st depression before 1st mania	55 (35.5)	89 (57.4)	–
Visual hallucinations ever	11 (7.7)	23 (15.0)	3.92
Auditory hallucinations ever	18 (12.5)	23 (15.0)	0.40
Delusions ever*	30 (21.1)	59 (39.1)	11.14
Alcohol use disorder*	17 (11.0)	52 (33.5)	22.84
Suicide attempt	23 (15.6)	26 (17.1)	0.12
Head injury	42 (61.8)	26 (38.2)	4.82

	Mean (SD)	Mean (SD)	*t* value
# Children*	3.1 (3.2)	1.5 (1.9)	−5.23
# School years*	9.8 (3.6)	15.5 (2.9)	15.23
Age illness onset	25.0 (10.2)	27.0 (12.0)	1.52
Age 1st depressive episode	21.9 (9.2)	23.4 (12.1)	1.14
Age 1st manic episode	24.7 (10.3)	26.0 (11.6)	0.87
# Psych hospitalizations	5.5 (9.1)	3.2 (3.4)	−1.92
# Illicit drugs used*	0.3 (1.0)	1.8 (2.3)	7.44
# Suicide attempts	2.8 (3.4)	2.9 (3.3)	0.10

* Bonferroni *p* < 0.002

Some differences in comorbidity were also observed (Table 1). Anabaptists reported fewer occurrences of medical illness, less alcohol and drug use, and were less likely to experience delusions outside of a mood episode. Interestingly, Anabaptists reported a higher lifetime incidence of head injury.

**Fig. 1:**
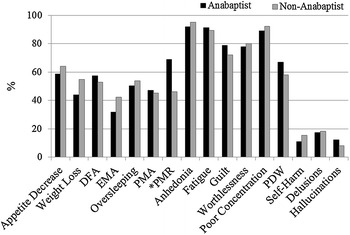
Symptoms reported during the most severe episode of major depression in Anabaptist and non-Anabaptist participants. *DFA* difficulty falling asleep, *EMA* early morning awakening, *PMA* psychomotor agitation, *PMR* psychomotor retardation, *PDW* passive death wishes; *Bonferroni *p* < 0.001

**Fig. 2:**
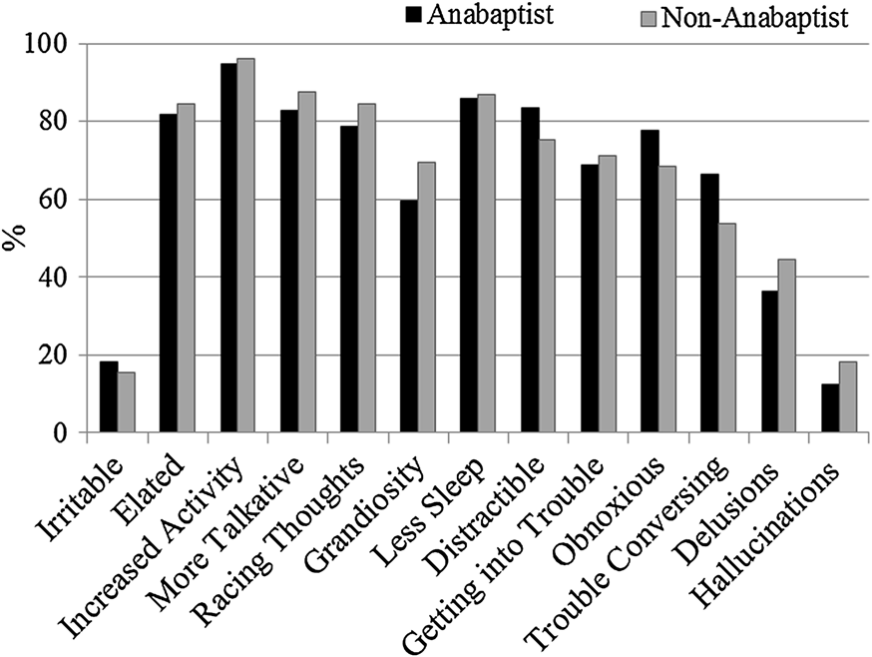
Symptoms reported during the most severe episode of mania in Anabaptist and non-Anabaptist participants

The symptom profiles for the most severe episodes of major depression (Fig. 1) and mania (Fig. 2) were very similar between the Anabaptists and non-Anabaptists. However, Anabaptist participants did report higher rates of psychomotor retardation during major depressive episodes (*χ* ^2^ = 14.47, Bonferroni *p* < 0.004).

### Factors influencing mood disorder severity

**Fig. 3:**
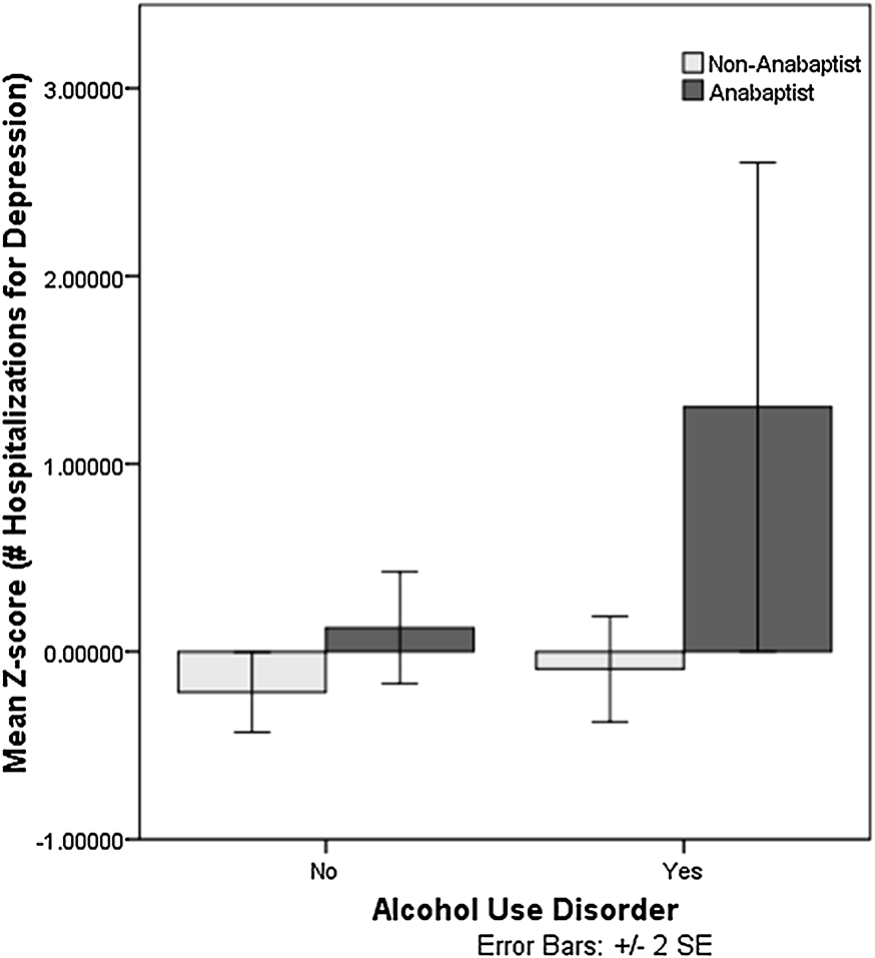
Main effects of group and alcohol use disorder on number of hospitalizations for major depression in Anabaptist (*n* = 49) and non-Anabaptist (*n* = 92) participants

**Fig. 4:**
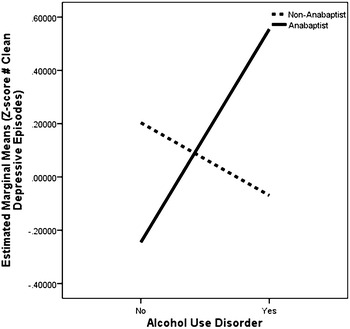
Interactive effects of group and alcohol use disorder on number of “clean” major depressive episodes in Anabaptist (*n* = 100) and non-Anabaptist (*n* = 113) participants

Alcohol use disorder (AUD) was associated with greater severity of major depression within Anabaptists, but not non-Anabaptists, in this study. AUD within the Anabaptists was associated with more hospitalizations for depression, *F*(1, 136) = 10.73, *p* = 0.001, corrected *p* = 0.015 (Fig. 3). There was also a significant interaction between group and number of “clean” depressive episodes (not directly associated with alcohol use): AUD was associated with more episodes among Anabaptists but fewer episodes among non-Anabaptists, *F*(1, 208) = 9.28, *p* = 0.003, corrected *p* = 0.045 (Fig. 4). There were no significant effects of AUD on age at first major depression, number of symptoms during the most severe episode of major depression, or frequency of major mood episodes in either group.

Lifetime history of head injury showed a trend toward association with more episodes of major depression in both Anabaptist and non-Anabaptist cases, *F*(1, 208) = 6.87, *p* = 0.009, but this main effect was not significant after Bonferroni correction. Out of 27 Anabaptists with a head injury, 17 reported their first head injury before onset of major mood disorder, with a mean interval between injury and onset of 8 years. Most of the reported head injuries were reported to be without loss of consciousness or need for medical attention. There were no significant effects of head injury on age at first major depression, number of symptoms during the most severe episode of major depression, number of hospitalizations for major depression, or frequency of major mood episodes in either group.

## Discussion

We sought to fill several important gaps in the literature by comparing the course and symptom profiles of major mood disorders between Anabaptists, a unique population characterized by varying degrees of cultural and genetic isolation, and cases drawn from the general population who were matched on age, gender, and diagnosis. Two main findings emerged: (1) Despite substantial cultural and genetic differences, manic and depressive symptom profiles were very similar among Anabaptist and non-Anabaptist participants. (2) Alcohol use disorders were associated with greater severity of major depression in the Anabaptist, but not the non-Anabaptist, participants we studied. The results demonstrate that the symptomatology of bipolar disorder and major depression shines through cultural and genetic differences, while alcohol comorbidity may differentially influence the course and severity of major mood disorders in important ways.

The findings of this study should be interpreted in the context of several limitations. Anabaptist participants were mostly recruited from psychiatric hospitals, which may oversample cases with more severe mood disorders within this population. Individuals who are ill enough to require hospitalization may not be representative of mood disorders in the community, especially where awareness of mental illness and access to mental health services are limited. Non-Anabaptist cases were previously enrolled on the basis of a diagnosis of bipolar I or schizoaffective bipolar disorder, and many were not ascertained in hospital settings (Potash et al. 2007). This ascertainment difference probably explains the higher overall rates of major role impairment, greater number of psychiatric hospitalizations, and increased use of psychotropic medications in the Anabaptist cases we studied. Ascertainment differences are unlikely to account for the observed effects of alcohol use or head injury, however, since those analyses were controlled for group differences in psychiatric hospitalization rates. Assessment procedures relied on the DIGS, which is a well-validated instrument (Nurnberger et al. 1994), but does not provide good quantitative measures of psychiatric symptomatology outside major DSM-based diagnostic categories. Our ongoing work in the Anabaptist communities incorporates additional measures of neurocognition (Cardenas et al. 2016) and subsyndromal symptoms, but these measures are not available for the non-Anabaptists in the Phenome Database (Potash et al. 2007).

Despite these limitations, several conclusions can be drawn. We found that the symptom profiles of severe major depressive and manic episodes were remarkably similar in the Anabaptists and non-Anabaptists we studied. These findings are in broad agreement with the previous studies of Egeland and colleagues performed in the Pennsylvania Amish (Egeland and Hostetter 1983; Egeland et al. 1983; Hostetter et al. 1983). Differences in rates of marriage, number of children, and education level were all expected based on the previous literature (Brewer and Bonalumi 1995; Kreps et al. 2004) and Anabaptist lifestyles and beliefs. The higher rate of psychomotor retardation reported by Anabaptist participants may reflect a greater awareness of this symptom owing to active lifestyles and widespread reliance on physical labor.

We found that alcohol use disorder (AUD) worsened the course of mood disorder, but only in the Anabaptist participants we studied. Prior work has shown that alcohol use increases incidence of major depression (Gilman and Abraham 2001). We ran an analysis of the entire Non-Anabaptist database (Phenome database *n* = 2897), and found that the presence of an AUD was related to increased depressive episodes, similar to previous findings. Therefore, our findings are likely the result of a gender distribution that differs from the overall Phenome sample. Gender distributions were significantly different in our matched sample compared to those in the full Phenome database (matched sample = 60 % male, overall Phenome database = 37 % male, *p* < 0.05). Therefore, the results should be interpreted with the greater proportion of females in mind.

Since there are more practical and cultural barriers to alcohol use among Anabaptists, AUD in this group may reflect poorer social adjustment and other psychosocial vulnerabilities that contribute to increased depressive episodes. AUD may also lower the threshold for psychiatric hospitalization among Anabaptists. We also observed that AUD was associated with increased number of clean depressive episodes among the Anabaptists we studied. This suggests that those Anabaptists who do develop AUD are vulnerable to increased depressive episodes, that depressive episodes predispose to AUD, or that some third factor—such as social isolation or deviance—is involved. Further studies that use a longitudinal design would be needed to clarify this association.

We found that the course and severity of mood disorder was associated with head injury at a trend level in both Anabaptist and non-Anabaptist participants, although head injuries were more common among Anabaptists. Previous studies have also found a higher rate of head injuries in Anabaptists. One study found that 76 % of injuries in Amish children aged 4 months to 18 years involved the head and central nervous system (Vitale et al. 2006). Previous work has also shown that head injuries are an important contributor to mood disorder morbidity (Mrazek and Haggerty 1994). Between 14 and 50 % of individuals who experience a traumatic brain injury develop a mood disorder within a year of the injury (Deb et al. 1999; Jorge and Robinson 2003). Our results are consistent with these findings, and highlight the potential importance of head injuries that might be considered “minor” since they do not lead to loss of consciousness or medical attention. If confirmed in larger samples, the results might suggest that public health measures aimed at reducing head injuries could have a beneficial impact on mood disorder morbidity.

## Conclusion

This is the first study to directly compare major mood disorders in Anabaptist and non-Anabaptist individuals. Despite cultural differences, there was a remarkable similarity in symptom profiles for severe episodes of mania or major depression. However, alcohol comorbidity, head injury, and their effects on illness course suggest some differences that deserve further investigation. These results also show that the presentation of a highly heritable psychiatric illness such as bipolar disorder does not differ much in the genetically isolated Anabaptist population, implying that genetic insights enabled by isolated populations might also inform our understanding of psychiatric disorders in the general population.

## Abbreviations

AUD: alcohol use disorder
AMBiGen: Amish Mennonite bipolar genetics study
ANCOVA: analysis of covariance
DIGS: diagnostic interview for genetic studies
DSM-IV: diagnostic and statistical manual of mental disorders, 4th Edition
NIMH: National Institute of Mental Health
SPSS: statistical package for the social sciences
